# Remembering Hirsh Cohen and His Role in Developing Computational Neuroscience

**DOI:** 10.1523/ENEURO.0284-22.2022

**Published:** 2022-07-29

**Authors:** L.F. Abbott, Eve Marder

**Affiliations:** 1 Zuckerman Mind, Brain and Behavior Institute, Columbia University, New York, New York 10027; 2 Volen Center and Biology Department, Brandeis University, Waltham, Massachusetts 02454

**Keywords:** computational neuroscience

We mourn the loss of Hirsh Cohen who died on December 20, 2020, at the age of 95. Many of us benefitted from Hirsh’s humor and wisdom, as he fostered the early growth and well being of the field of computational neuroscience. When we first met Hirsh, he was just starting his second full career, as a grant officer at the Sloan Foundation. (In his first career, Hirsh was an applied mathematician in academia and at the IBM Research Laboratory). In 1994, the field of computational neuroscience was small, and Hirsh felt strongly that bringing young mathematicians, physicists, and computer scientists into neuroscience would energize and expand the field. Consequently, Hirsh established five Sloan Centers to fund postdocs who wished to transition from previous training in the physical sciences into neuroscience. Hirsh insisted that the Sloan Centers be led by experimentalists to ensure the full integration of the first cadres of postdocs into their new field. This initiative brought over 100 postdocs into neuroscience, many of whom have become world leaders. The Sloan Foundation, like many other foundations, believed that their role was to initiate programs and then to move on to new ventures. Hirsh believed so strongly in computational neuroscience that he worked to establish a collaboration with the Swartz Foundation, which today maintains the funding and philosophy of the original program that Hirsch began.

Hirsh was so much more than a grant officer. He cared deeply about mathematics and neuroscience and he made strong connections with the postdocs he funded, becoming a surrogate academic grandfather to many of this new generation. He encouraged them to think big and excel. At the same time, he gave wise guidance to senior leaders in the field and served to maintain a kind of moral compass that held senior and junior scientists to high standards. Hirsch also facilitated the establishment of the yearly CoSyne meeting, now one of the most important scientific meetings in systems neuroscience. Eventually, Hirsh retired from the Sloan Foundation, but for many years he was a senior advisor to the Swartz Foundation, where he continued to foster the careers of multiple generations of postdocs.

In addition to his impact on computational neuroscience, Hirsch was instrumental in establishing the Sloan Digital Sky Survey, a groundbreaking map of large-scale structure in the universe. In all his endeavors, Hirsh pushed hard for top quality science, but he never lost sight of the fact that science is done by people, and his interest and pride in the trainees he sponsored inspired all of us. We still feel his presence, urging and encouraging us to apply mathematical tools to the mysteries of the brain.

